# Insulators in Plants: Progress and Open Questions

**DOI:** 10.3390/genes12091422

**Published:** 2021-09-16

**Authors:** Amina Kurbidaeva, Michael Purugganan

**Affiliations:** Center for Genomics and Systems Biology, New York University, New York, NY 10003, USA; mp132@nyu.edu

**Keywords:** insulator, chromatin, genome organization, architectural proteins

## Abstract

The genomes of higher eukaryotes are partitioned into topologically associated domains or TADs, and insulators (also known as boundary elements) are the key elements responsible for their formation and maintenance. Insulators were first identified and extensively studied in Drosophila as well as mammalian genomes, and have also been described in yeast and plants. In addition, many insulator proteins are known in Drosophila, and some have been investigated in mammals. However, much less is known about this important class of non-coding DNA elements in plant genomes. In this review, we take a detailed look at known plant insulators across different species and provide an overview of potential determinants of plant insulator functions, including cis-elements and boundary proteins. We also discuss methods previously used in attempts to identify plant insulators, provide a perspective on their importance for research and biotechnology, and discuss areas of potential future research.

## 1. Introduction

Insulators, or boundary elements, are non-coding *cis* regulatory elements that are important for proper gene regulation and the maintenance of chromosome organization. Insulators have two major functions related to gene regulation: enhancer-blocking (preventing unwanted enhancer–promoter interactions) and barrier functions (stopping the spread of repressive or active chromatin along the chromosome). These activities have been identified through transgene assays in model organisms. On a global scale, insulators organize the chromosome into a series of discrete regions. Mechanistically, pairs of insulators interact with each other to generate chromosomal loops and form the basis of topologically associated domains (TADs). Two key elements are necessary for the proper functioning of insulators: (1) the presence of certain DNA motifs, acting as binding sites for (2) specific proteins known as insulator, boundary, or architectural proteins.

## 2. The Discovery of Insulators and Their Key Characteristics

A “boundary model” of chromosome structure was first proposed as a result of studies of the mechanisms of heterochromatin formation in Drosophila. The model postulated that there are certain terminating sites, or boundaries, on the chromosome that restrict the propagation of heterochromatic structures [1]. These chromatin boundaries, or insulators, were first identified in Drosophila [2] as *scs* and *scs’* (specialized chromatin structures), which flank the two *hsp70* genes at cytogenetic locus 87A7. These fragments were described as nuclease-hypersensitive regions of DNA arranged around a central nuclease-resistant segment, and were shown to be anchor points for the domain organization of this region. By definition, *scs* and *scs’* insulators are barrier insulators, because they protect the locus from the spread of heterochromatin. However, they also prevent the interaction between an enhancer and promoter in an enhancer-blocking assay. Thus, they perform both enhancer-blocking and barrier functions. Subsequently, many other insulator elements in Drosophila were identified and studied, including *gypsy* [3], *Bithorax* complex [4,5,6], and *even-skipped* gene insulators [7]. Some of the insulators identified were shown to have both functions, similar to *scs* and *scs’*, while others only had an enhancer-blocking or barrier function. However, recent studies suggest that these two functions are probably inseparable and might be conferred by the same mechanism, namely, the formation of a chromatin loop by a pair of insulators. The mechanics of this process have been best studied in flies [7]. Similarly, CTCF-dependent insulators in mammals are also thought to perform both blocking and barrier functions [8].

The study of fly insulators helped establish the key principles underlying insulator function. First is the enrichment for specific DNA sequences or protein-binding sites [9]. Indeed, these clusters of recognition motif sequences can be used as a boundary-prediction tool in Drosophila [10].

Second, pairing interactions between insulators are crucially important for their function. According to the now widely accepted looping model, insulators do not act individually, and instead two insulators engage in long-distance interactions with the help of insulator binding proteins, creating a chromatin loop. The sequences within the loop, such as enhancers and promoters, can engage in regulatory interactions, and sequences outside of the loop do not interact with the sequences within the loop.

Third, insulator sequences exhibit a preference for certain partners, dictated by the proteins binding to sites within an insulator. These interactions can be homologous or heterologous; however, they are always specific [9].

Fourth is the locus-specific composition of proteins binding to insulator DNA. Architectural proteins are a heterologous class of proteins, most of them with a DNA-binding domain. These domains include zinc finger domain proteins (fly Su(Hw), Zw5, dCTCF, GAF, Mod(mdg4)-67.2, CP190, ZIPIC, Pita, vertebrate YY1 protein, CTCF), BED finger domain (fly BEAF-32), and BEN domain (fly Elba1, Elba2) proteins [11,12]. Several proteins previously known for other functions in the genome have been implicated in insulator function in recent years, such as Clamp [13], Nup98 [14] and Insv [15]. Some proteins without DNA binding activity, such as fly Chriz and enhancer of yellow 2, are also involved in insulator functioning [16], and appear to act by engaging in interactions with DNA-binding proteins.

Finally, interactions between insulators are orientation dependent. Insulators are essentially an array of binding sites, and architectural protein–protein interactions promoting insulator function are possible when binding sites are oriented in a certain way [7,9].

There are some additional important features ascribed to insulators. Insulators are relatively short (100–2000 bps) DNA fragments, with accessible chromatin, as evidenced by their sensitivity to DNase digestion [17]. They are frequently located between divergent promoters, and are associated with activating chromatin marks such as H3K4me3 and H3K9ac [18]. For this reason, promoters frequently score as insulators in classical insulator assays when an element is placed between an enhancer and a promoter [19,20]. Indeed, insulators are thought to have originated from promoters, and during evolution insulators may have acquired the ability to bind proteins capable of remodeling chromatin by removing nucleosomes, and are able to mediate long-range interactions [21].

A number of studies suggest that in Drosophila, there may be a direct relationship between the ability of a DNA sequence to act as an insulator in enhancer-blocking assays and to be a TAD boundary. High-resolution chromosome conformation capture (Hi-C)-based studies show that boundaries between topological domains map onto classical insulator elements [22,23]. TAD boundaries in Drosophila are also enriched in architectural protein binding sites, and they also function as enhancer blockers in transgenic assays [17,23]. Insulators also show evidence of local sequence constraint [24], and the level of conservation of insulator sequences positively correlates with their strength as boundaries [25]. Reporter-based assays show that insulator activity (e.g., “boundary strength”—the ability of an insulator to effectively block interactions between loci located on either of its sides) is positively correlated with the occupancy level of architectural protein binding sites, suggesting co-binding by architectural proteins underlies the functional potential of these loci [17].

## 3. The Existence of Plant Insulators

The assumption that plants might also have insulators comes from the following observations. The first observation is that the principles of genome organization are evolutionarily conserved in Eukarya, and insulators have been identified across animal and fungi kingdoms. Many functional insulators have been identified within the genus Drosophila and in vertebrates (reviewed in [26]), and even more species from diverse clades of eukaryotes have homologs of insulator proteins (reviewed in [11]). In addition to fly and mammalian genomes, functional insulators have also been found in sea urchins [27] and yeast [28]. The widespread presence of insulators also arises from Hi-C analyses of eukaryotic genomes, which offer data on the protist *P. falciparum*, the fungus *N. crassa*, and a number of animals (*C. elegans*, *D. melanogaster*, *H. sapiens*, *M. musculus* and others) [29].

Plant genome organization seems to be governed by the same principles as are observed in other eukaryotic systems, where chromatin loops, TADs, and compartments can be identified by analyzing Hi-C contact maps. To date, such maps have been reported for genomes of *A. thaliana* and *A. lyrata*, *Brassica rapa* and *B. oleracea*, rice, soybean, maize, poplar, cotton, foxtail millet, sorghum, tomato, and a non-vascular plant *Marchantia polymorpha* [30,31,32,33]. Except for the two Arabidopsis species, TADs have been reported in all the plants studied. This might indicate the presence of insulators at TAD borders, similarly to animal genomes. However, there is no clear evidence as to the nature of plant TADs, and future research is required to examine this issue. In Arabidopsis, despite its genome not having clear TADs, over 1000 TAD-boundary-like and insulator-like sequences were found [30]. These sequences possess similar properties to those of animal TAD borders/insulators—chromatin contacts crossing insulator-like regions are restricted, and they are enriched for open chromatin.

A second important observation comes from experiments where insulators from a variety of species have been tested and shown to be functional in plants. This suggests that the components involved in insulator activity in these organisms are evolutionarily conserved, and that some proteins or protein complexes in plant cells recognize the motifs within foreign insulator sequences, bind to them, and exert an insulator function. Non-plant insulator sequences that, at least to some extent, act as insulators in transgenic Arabidopsis include UASrpg isolated from *Ashbya gossypii*, BEAD1c from *H. sapiens* [34], and Fab-7 and gypsy from Drosophila [35,36]. In transgenic tobacco, an insulator from the sea urchin *Hemicentrotus pulcherrimus* exhibited enhancer-blocking activity [37]. The notion that insulator function is conserved across kingdoms is further supported by the finding that binding sites for insulator proteins Rap2, CTCF and Su(Hw) were functional in transgenic Arabidopsis as a part of the insulator heterologous sequences from *A. gossypii*, *H. sapiens* and *D. melanogaster.*

Third, the existence of insulators is supported by the results of experiments with transgenic constructs bearing strong constitutive promoters that were inserted into plant genomes. In such experiments, the CaMV 35S enhancer in the construct does not always increase the expression of nearby endogenous genes, as would be expected if no chromatin barriers were restricting enhancer function. In one study in rice, for instance, only 4 of the 10 genes in which constructs were inserted within 4.5 kb displayed elevated expression, with no enhanced expression observed in the remaining lines. In the latter cases, the explanation is that the presence of an as-yet unidentified insulator element prevented the misregulation of genes by restricting the effects of enhancer elements to specific domains [38].

The underlying principle governing insulator function is the establishment of chromosomal interactions that induce different sequences in close proximity within the nucleus, which leads to a variety of outcomes, such as blocking repressive chromatin, restricting enhancer–promoter interactions, and the establishment of TADs. While it is possible that some mechanisms/factors emerged early in evolution and are now shared between lineages, it is also likely that different eukaryotic lineages have evolved unique mechanisms or factors to achieve insulator function. For example, in animals, the CTCF and GAF proteins have a conserved architectural function between Drosophila and mammals [39,40]; nevertheless, additional unique architectural proteins exist in the Drosophila genome [16]. Thus, there are three possible scenarios for the evolution of the chromatin architecture in plant genomes. We can expect plants to either (1) have only unique architectural proteins (compared to animal genomes), (2) to have homologs of known animal proteins involved in their insulator function, or (3) have a combination of (1) and (2).

## 4. Experimentally Verified Plant Insulators

A classic method to identify insulator activity in a genomic fragment is an enhancer-blocking assay on transgenic lines, first established in Drosophila [19]. This assay is used to define an insulator as an element that can block the activity of an enhancer when positioned between it and a promoter. Later “insulator bypass” experiments, where a pair of insulators was located between an enhancer and a promoter, showed that they “neutralized” each other and could not block enhancer–promoter interaction [9]. These and other experiments, including fluorescent microscopy studies, have led to the acceptance of a looping model of insulator action, which postulates that an insulator needs a partner to perform its enhancer blocking functions.

Based on this evidence, the necessary assay for an insulator function is a dual insulator assay, where a pair of insulator elements (homologous or heterologous) brackets an enhancer or promoter; we will discuss this later in this review. Nevertheless, the classic enhancer-blocking assay has remained the method of choice to prove a sequence has insulator functions, and it has been modified to verify the insulator-like activity of candidate plant sequences in Arabidopsis, tobacco, and rice. To test for insulator activity in Arabidopsis, for example, a candidate insulator sequence was placed between CaMV 35S enhancers and a specific promoter that drives expression of the reporter gene β-glucuronidase; promoters used include the seed-specific *NAPIN* promoter [35], the *AGAMOUS* promoter [41,42], and the *PISTILLATA* promoter [43,44]. The expression pattern of the reporter gene was then assessed using histochemical GUS staining in leaves and inflorescences of transgenic plants, and a number of sequences have been identified as plant insulators on the basis of this and similar assays.

From these assays, a handful of plant-derived insulator sequences have been found across plant species. These include the NI29 insulator from Arabidopsis and its derived version M4 [45]. NI29 includes a small 16-bp palindromic sequence, which when positioned between the 35S enhancer and a core minimal promoter blocks the enhancer function in transgenic plants. Interestingly, the M4 element, an artificially mutated version of NI29, doubles the activity of its native precursor [45]. However, a different study could not demonstrate the 35S enhancer-blocking activity of NI29 [42], and has even been found to enhance the misexpression of the transgene [35]. This inconsistency may indicate the importance of the surrounding genetic context in the proper functioning of insulators. When an insulator is not paired with a partner sequence, it will pair with the nearest compatible element in proximity to the locus, creating unwanted effects.

Another candidate plant insulator is the *gypsy*-like sequence found in the Arabidopsis genome, which displayed enhancer blocking effects when inserted between the 35S enhancer and Pip (petal- and stamen-specific *PISTILLATA* promoter) [44]. One insulator-like sequence has also been identified in the *HSP70* gene promoter of rice. This sequence, named HS185, was also able to block the activity of the 35S CAMV enhancer [46].

A well-studied class of putative insulator elements with potential applications in plant transgenic technology are the matrix or scaffold attachment regions (MARs/SARs), which are suggested to promote the formation of chromatin loops and exhibit enhancer-blocking activities. MARs are non-coding AT-rich sequences that preferentially bind to the isolated nuclear matrix in vitro [47]. When flanking a transgene, MARs have been shown to reduce the chromosomal position effects, which results in an increase in the level of transgene expression and/or a reduction in plant-to-plant variability in reporter gene expression [48]. An example of MARs is a 2-kb region called the transformation booster sequence (TBS) in *Petunia hybrida*, which was found to function as an enhancer-blocking insulator when inserted between the CaMV 35S promoter/enhancer and an AGIP promoter (one of the *AGAMOUS* gene promoters) driving the expression of the *GUS* (*β-glucuronidase*) gene in Arabidopsis [49]. A tobacco MAR, TM2, has also been shown to minimize transgene silencing by nearby chromatin when placed upstream and downstream of a transgene [50]. Two SARs from the chickpea *Cicer arietinum* (SAR1 and SAR2) were able to protect transgenes from position effects in transgenic tomato and chickpea [51]. Similar insulator-like properties are also exhibited by the P1-SAR and *Gmhsp* 17.6L MAR of soybean, *Rb7* 3′ MAR of tobacco, *Adh1* 50 MAR of maize, *b-phaseolin* 5′ and 3′ MARs of *P. vulgaris*, and the plastocyanin gene 3′ MAR of pea [48].

## 5. Plant Boundary Proteins

As mentioned earlier, the activity of insulators from *A. gossypii*, *H. sapiens* and *D. melanogaster* is dependent on the presence of binding sites for insulator proteins Rap1, CTCF and Su(Hw) in transgenic Arabidopsis. However, homologs of CTCF or Su(Hw) have not been found in plant genomes, including Arabidopsis [36,52]. Nevertheless, clearly, some proteins are able to bind to this insulator DNA in Arabidopsis cells, and future studies will hopefully identify these factors.

Nevertheless, several transcription factor families have been implicated in plant TAD boundary formation based on the motif enrichment profiles of DNA sequences at TAD borders. In rice, TAD boundary sequences are enriched for binding motifs for TCP class I (*TEOSINTE-LIKE1, CYCLOIDEA,* and *PROLIFERATING CELL FACTOR1*) and bZIP (basic leucine zipper) transcription factors [18,32]. TCP proteins are plant-specific DNA-binding transcription factors, and in total, more than 20 TCP genes are found in plant species [53]. Interestingly, a TCP1 protein was found at the TAD boundaries in the basal plant Marchantia, suggesting the evolutionary conservation of architectural functions of TCP family proteins [33]. However, the loss of TCP1 did not result in changes in genome architecture in Marchantia, and there may be functional redundancy between architectural proteins, similar to that observed in Drosophila [16,54]. In addition, TAD borders in Marchantia are enriched in BBR/BPC and bHLH TF family binding sites, implying that proteins binding to these sequences might also contribute to boundary formation. BBR/BPC proteins are plant-specific TFs recognizing (GA/TC)n repeats, found to be enriched in promoter regions in the Arabidopsis genome [55]. Interestingly, GA repeats are recognized by the GAF protein in Drosophila, which has an architectural function [9]. Thus, binding sites may be conserved between kingdoms, but the proteins recognizing these sites might have evolved independently. In the Marchantia genome, there is a BBR/BPC homologue, *Tak-1* [33], which may be a good candidate for an architectural protein. However, the architectural role for these proteins has been suggested but not demonstrated, and further studies are required to verify the functions of these proteins in shaping genome architecture.

Recently, a three-member family of putative negative transcription elongation factors from Arabidopsis, named the BORDER proteins (BDR1, BDR2, and BDR3), has been described as the first plant proteins with insulator protein-like activity [56]. BDR proteins contain a SPOC domain found in the SPEN family of transcriptional repressors, and a central domain of the transcription elongation factor IIS (TFIIS). These proteins are enriched in intergenic regions and inhibit transcriptional interference by promoting 3′ pausing in upstream genes, thus protecting the promoter of a downstream gene from invasion by Pol II. In addition, upstream tandem neighbors of BDR-protected genes are enriched in gene loops, which suggests that BDR may prevent transcriptional interference by altering chromatin architecture. ChIP-seq data showed that BDR peaks are located in nucleosome-depleted, DNase-hypersensitive regions, and contain evolutionarily conserved TCP-like and E-box motifs. BDR proteins lack characterized DNA-binding motifs, so they are likely recruited to chromatin by other factors, such as TCP proteins. Using methods to detect DNA–protein complexes, such as chromatin immunoprecipitation (ChIP), DNA electrophoretic mobility shift assays (EMSA), or yeast one-hybrid (Y1H) assays, may be necessary in the future to identify plant insulator proteins.

## 6. Genome Organization in Animals and Plants and the Function of Insulators

In recent years, the whole-genome analysis of chromatin conformation has allowed for the identification of the basic principles of genome organization. Hi-C, and its derivative Micro-C [57], have been extensively applied to the study of animal genomes and some plant genomes, mostly in Arabidopsis [29]. There appears to be differences in the organization of plant and animal genomes, and it seems that insulator elements have evolved to have slightly different functions in these kingdoms, despite possible similarities in the mechanism of their function.

An interesting difference between the Arabidopsis and animal genomes was pointed out in [56]. In animals, enhancers are often located many kilobases away from their target promoter [58], which creates a problem regarding how to ensure an enhancer element associates with the promoter of the correct gene while not acting on promoters of other nearby genes. It seems that in animals, insulator proteins help to solve both problems through the formation of loops/TADs, which allows enhancers to be “insulated” from genes outside the loop. In Arabidopsis, however, most enhancer sequences are located close to the promoter [59], and insulators and TADs are less needed. However, a different problem exists in the relatively compact Arabidopsis genome—transcriptional interference between closely spaced genes. The solution to this problem may also be chromatin loops, formed by the interactions between insulator DNA and proteins.

The genome structure of two Arabidopsis species—*A. thaliana* and *A. lyrata*—appears to differ from the genome organization of other plants, as determined by Hi-C contact maps. Prominent TADs have been detected in the genomes of rice, maize, barley, and other cereal crop species [32]. *A. thaliana* and *A. lyrata*—plants with the smallest genomes among the plant species investigated—have no discernible TADs. Arabidopsis, however, has a genome size similar to that of Drosophila, but these species differ in gene activity profiles. In Drosophila, gene transcription levels vary throughout the genome: regions of high expression are interspersed by non-transcribed regions, whereas Arabidopsis gene expression is constant throughout the genome [29]. This observation might explain the seeming lack of TADs in the Arabidopsis genome. Alternatively, this lack of TADs in Arabidopsis may be due to the currently available resolution of the Hi-C approach. In animals, increasing the resolution of Hi-C from megabase to subkilobase scales leads to the detection of smaller TADs, which in Drosophila often span only one or two genes [60]. The introduction of Micro-C technology in recent years has allowed us to reach a nucleosome-scale resolution in chromosome contact maps, and unprecedented coverage [57]. It is possible that the higher density of genes with various expression levels increases the need for smaller TADs in the Arabidopsis genome, and implementing Micro-C technology may help detect possible small Arabidopsis TADs.

## 7. Insulators in Biotechnology

Transgenic plants have been a major source of agricultural innovation as part of a technological toolkit to ensure future food security. The classic method of generating transgenic plants via Agrobacterium-mediated transformation, however, has its limitations, which stem from the random nature of transgene insertion. Transgenic DNA may integrate into regions of the genome that are transcriptionally repressed (heterochromatin), which can result in transgene silencing, or it may integrate near strong enhancers/silencers, which could lead to unwanted expression patterns in the transgene. Conversely, regulatory elements within the transgene may influence endogenous genes near the site of insertion. Due to the unpredictable nature of transgene expression in plants, one of the most important technical challenges with regards to plant biotechnology is the development of methods to mitigate transgene interference. Even with the advent of CRISPR technology, when the locus of gene insertion is non-random, it will still be important to protect the gene from expression interference.

A number of enhancer-blocking insulators have been shown to reduce enhancer–promoter interference and chromosomal position effects in transgenic plants (for a comprehensive review, see [61]). The insulators and insulator-like sequences that have been used can protect a transgene from unwanted interactions with endogenous genetic elements, including many from non-plant species (*A. gossypii*, human, chicken, sea urchin and Drosophila), as well as plant-derived sequences (P. vulgaris, pea, Arabidopsis, maize, tobacco, soybean). These sequences were used to protect engineered transgenes in a variety of plants, including Arabidopsis, tobacco, maize, rice, barley, cacao, poplar, and Brassica [61,62].

Possibly the best studied insulator-like elements with potential biotechnological applications are the MARs/SARs. Canonical MARs do not possess enhancer-blocking activity, and canonical insulators are not MARs. Therefore, we only call them insulator-like on the basis of their ability to engage in spatial interactions, forming chromatin loops similarly to insulators [63]. Using MARs to flank transgene insertions resulted in an increase in the level of transgene expression or a reduction in plant-to-plant variability [64]. Results using the same sequences in different species or with different promoters, however, showed mixed results. For instance, one of the MARs from upstream of the chicken lysozyme gene was able to protect GUS gene expression driven by the CAMV 35S promoter, but not by the NPTII gene; moreover, it worked only in tobacco plants and not in Arabidopsis [64]. A recent study compared the effects on the expression levels and variability of a transgene in Arabidopsis using a MAR located next to the tobacco root-specific gene *Rb7*, the chicken lysozyme A MAR region (chiMARs), the petunia transformation booster sequence (TBS) and one of the scaffold/matrix attachment region sequences isolated from Arabidopsis chromosome 4 (AtS/MAR10) [65]. All these sequences, when used as a pair flanking the transgene, were able to increase the expression of the transgene in Arabidopsis. However, in contrast to previous studies, neither chiMARs, Rb7 nor TBS reduced between-line or between-individual variation; in fact, they showed increased variability, which was attributed to the use of a different promoter controlling the transgene. These studies show that the effect of each insulator is different, and their use in transgenic constructs should depend on the needs of each specific experiment. These studies do show the potential of insulators for plant biotechnology applications, but to fully unleash this potential, we need to identify and study plant-specific insulators in detail.

## 8. Strategies Used to Identify Plant Insulators

Three main strategies have been utilized to search for plant insulators, with the first two strategies relying mostly on chance. The first strategy involves the production and subsequent screening of a random oligonucleotide library [66], in which candidate sequences were tested for insulator activity in Arabidopsis when inserted into vectors with different combinations of enhancers, promoters, and reporter genes. A second strategy involved the dissection of a rice *HSP70* gene [46]; here the discovery of an insulator sequence was a byproduct of promoter deletion analysis. The third strategy was based on sequence homology to the known Drosophila gypsy insulator, which led to the successful identification of an Arabidopsis gypsy-like insulator [44]. However, this approach would overlook insulators comprising only plant-specific architectural proteins’ binding sites.

What is required is a more systematic strategy for insulator identification that incorporates the features that have been thus far identified in other eukaryotic systems. There have indeed been efforts to establish a computational approach for insulator identification in animal species. There are algorithms that predict the locations of human TAD boundary elements based on the genomic distributions of chromatin and transcriptional states, utilizing DNA sequence information coupled with ChIP-seq data for architectural proteins, histone modifications and RNA Pol II-binding [67,68,69]. Another method, called pTADS (prediction of TAD boundary and strength), was able to predict TAD boundaries in multiple human cell lines from DNA sequence and epigenetic profile information, and the results were validated by Hi-C experiments [70]. However, the boundaries predicted by these methods have not been tested experimentally for insulator function. Machine learning methods have also been implemented for boundary prediction in human and Drosophila based on data from Hi-C and/or ChIP-seq experiments [71,72].

Another approach, named the chromatin domain boundary element search tool (cdBEST) [10], uses known recognition sequences of insulator proteins and searches for “motif clusters”. Using this approach across 12 Drosophila genomes allowed for the identification of thousands of candidate insulators, of which 80% were indeed functional as boundaries *in vivo*. However, ChIP-seq-based strategies can only predict potential insulators in organisms where insulator proteins’ binding sites are known and well-studied, and to date, the only genus for which this is possible remains Drosophila. Going forward, it will be necessary to accumulate knowledge about plant insulator proteins and their binding sites in order to develop efficient computational tools for candidate insulator identification.

## 9. Open Questions

The nature and function of plant insulators remains a mystery. We do not know how insulators function on a mechanistic level. What are the sequence determinants of plant insulators? What are the proteins that bind to them? Animal architectural proteins do not have homologues in plants, but certain DNA motifs seem to be conserved—hence, the question arises: does convergent evolution lead to the existence of sets of structurally different proteins, which nevertheless have similar functions? What are the rules governing the formation of chromatin loops? What is the basis for insulator partner choice—is it based on the distance between insulators that create a loop, or is the partner preference based on sequence and epigenetic determinants?

Another interesting question concerns the role of insulators in shaping the global architecture of the genome. Chromosome conformation studies revealed several key similarities, as well as some differences, in genome organization between plants and animals. Are these similarities and differences determined by the insulators structure and function—and if yes, can we find a set of characteristics distinguishing plant and animal insulators? To answer those questions, it is necessary to have an expanded set of experimentally verified insulator sequences. There are only a handful of sequences identified to date, and a major challenge is to find an effective means for the identification of plant insulators. The application of high-resolution 3C-derived technologies to study plant genomes may help identify and dissect insulator sequences.

These big questions are further aggravated by the technical challenges that plant biologists face in their search for plant insulator elements. The first issue concerns the choice of assay system and experimental design for the verification of insulator function in vivo. In several cases described in the literature, a certain sequence with insulator activity in one context failed in a different study, or when a different construct design or test organism was used (e.g., NI29, chiMARs, Rb7, TBS). This could be due to the fact that without a suitable nearby partner, potentially strong insulators could give a false negative result in classical enhancer-blocking assays. In accordance with the looping model of insulator function, classic enhancer-blocking assays with one insulator placed between an enhancer and a promoter may not be suitable for proving the insulator function of a sequence [9]. We think that establishing an assay system with two insulators would be necessary for the comprehensive assessment of insulator function in plants. In addition, it is possible that a loss of reporter gene activation is due to the putative insulator sequence being a repressor element. Adding additional layers of knowledge on chromatin signatures and insulator protein binding sites associated with insulators would greatly increase the validity of enhancer-blocking assays.

In addition to insulator sequences, the study of boundary proteins in plants is also an area that requires extensive research. Studies on Marchantia and rice suggest the presence of a redundant set of insulator proteins in plants. One of the more efficient methods of insulator protein identification in Drosophila is based on the search for position effect modifiers. In Arabidopsis, the existence of position effects has also been described. In a panel of transposon insertion lines generated in Arabidopsis, 20% showed up to 30-fold transgene expression differences [73]. Screens for the position effect modifiers in Arabidopsis and other plants would be a promising molecular genetic strategy for the identification of plant insulator proteins.

## 10. Concluding Remarks

Insulators are interesting sequences because of their fundamental role in shaping plant genomes, their contribution to gene regulation, and their potential use in transgenic plant technology. To date, there is only limited information on plant insulators and their associated architectural protein factors. The advent of new methods to examine genome organization, as well as the knowledge of insulators in other systems such as Drosophila, will hopefully spur greater efforts in identifying and studying plant insulators.

## Data Availability

Not applicable.
